# Economic effects of healthy ageing: functional limitation, forgone wages, and medical and long-term care costs

**DOI:** 10.1186/s13561-023-00442-x

**Published:** 2023-05-10

**Authors:** Shohei Okamoto, Haruka Sakamoto, Kazuki Kamimura, Kohei Komamura, Erika Kobayashi, Jersey Liang

**Affiliations:** 1 Research Team for Social Participation and Healthy Aging, Tokyo Metropolitan Institute for Geriatrics and Gerontology, 35-2 Sakae- cho, Itabashi-ku, Tokyo, 1730015 Japan; 2 Institute of Global Health Policy Research, National Centre for Global Health and Medicine, Tokyo, Japan; 3 Research Center for Financial Gerontology, Keio University, Tokyo, Japan; 4 Department of Hygiene and Public Health, Tokyo Women’s Medical University, Tokyo, Japan; 5 Hirao School of Management, Konan University, Hyogo, Japan; 6 Faculty of Economics, Keio University, Tokyo, Japan; 7 Department of Health Management and Policy, School of Public Health, University of Michigan, Michigan, USA; 8 Department of Health Care Management and Healthy Aging Research Center, Chang Gung University, Taoyuan, Taiwan

**Keywords:** Economic benefits, Disability, Functional health status, Successful ageing, Retirement, Medical care costs, Long-term care costs, D1, I1, J14, J26

## Abstract

This study aims to estimate the potential economic benefits of healthy ageing by obtaining estimates of the economic losses generated by functional limitations among middle-aged and older people. Utilising two data sources retrieved from nationally representative samples of the Japanese people, we analysed the association between functional limitation and economic indicators, including labour market outcomes, savings, investment, consumption, and unpaid activities among individuals aged ≥ 60. Using the estimated parameters from our micro-econometric analyses and the official statistics by the Japanese government and a previous study, we calculated the financial costs that can be averted if healthy ageing is achieved as foregone wages and formal medical/long-term care costs incurred by functional limitations. Our micro-econometric analyses found that functional limitation was associated with a 3% point increase in retirement probability, with a stronger association among those aged 60–69. Moreover, functional limitation was linked with higher total health spending and less active involvement in domestic work. Foregone wages generated by functional limitation were estimated to be approximately USD 266.4 million, driven mainly by individuals in their 60s. Long-term care costs, rather than medical care costs, for older people aged ≥ 85 accounted for most of the additional costs, indicating that the estimated medical and long-term costs generated by functional limitations were approximately USD 72.7 billion. Health interventions can yield economic benefits by preventing exits from the labour market due to health issues and reducing medical and long-term care costs.

**Supplementary Information:**

The online version contains supplementary material available at 10.1186/s13561-023-00442-x.

## Introduction

### Population ageing and disease burden

Worldwide, the ageing rate of people aged ≥ 65 in 2020 was 9.3%, and it may rise to 18% by 2060 and 23% by 2100 [1]. Japan, in particular, had an ageing rate of 28% in 2020, which will reach its peak of 38% in 2060 and then moderately decrease to 37% in 2100. Population ageing is not always negative, meaning that countries can achieve longevity due to many successes, such as economic growth, nutritional and hygiene improvements, medical development, and progress towards universal health coverage. However, many countries experience a gap between life expectancy and healthy life expectancy, mainly due to longevity itself and functional limitations caused by non-communicable diseases (NCDs) [2]. Despite the disparities across regions in life and healthy life expectancy at birth, populations in most countries spend around 10 years in poor health [2]. Therefore, reducing the disease burden arising from functional limitations and other comorbidities through effective interventions is essential to ensure that everyone can live healthily.

### Economic return on health investment

Many studies, most of which are based on the value of statistical life approach, suggest that health interventions, such as immunisation, tobacco and alcohol controls, and nutritional intervention for both infectious and non-communicable diseases, can produce economic benefits [3–9]. For instance, the most efficient package of interventions for NCDs can avert 39 million deaths in all low- and middle-income countries with an additional cost of USD 140 billion from 2023 to 2030, resulting in an economic benefit of USD 2.7 trillion (benefit-cost ratio = 19:1) [5]. Delayed ageing, which reduces mortality and the probability of onset of both chronic conditions and functional limitation, generates larger economic values through statistical life and health/social expenditures than merely extending life expectancy without an increase in healthy life expectancy and combating individual diseases [10, 11].

In many industrialised countries, responding to financial challenges associated with population ageing, particularly healthcare and long-term care costs, is an important policy target. Some policymakers and scientists believe that this could be addressed by extending healthy life expectancy and achieving healthy ageing. However, whether health improvement by intervention is a valuable tool to accomplish this policy target remains controversial because many of the interventions may be cost-effective but not cost-saving [12]. Since healthcare is designated to improve or maintain health but not to save money, the economic benefits of health investment may be underestimated or mismeasured if emphasis is given only to healthcare costs as the financial outcomes of the investment [13]. With a more comprehensive range of economic indicators (e.g., labour market outcomes, non-health consumption, and unpaid productive activities), returns on investments in health can be larger.

However, few studies have comprehensively assessed the association between healthy ageing and these economic indicators. Moreover, the potential economic benefits of healthy ageing are less known. Only two studies in the United States estimated the potential economic gains of healthy ageing as the statistical value of life by a willingness-to-pay approach [10] and using the costs of major entitlement programs (e.g., Medicare and Medicaid) [11].

### The linkage between health and economy among older adults

Health is an important element of human capital, which determines one’s productivity, time available for economic activities, and utility [14, 15]. At a macroeconomic level, health (i.e., life expectancy) is positively associated with countries’ economies and growth [16, 17]. In population ageing, a decline in the labour force will reduce economic growth [18]; however, this decline can be mitigated if the middle- and old-age population remains in good health [19]. Therefore, the health status of the population is an important driver of countries’ economies.

**Fig. 1:**
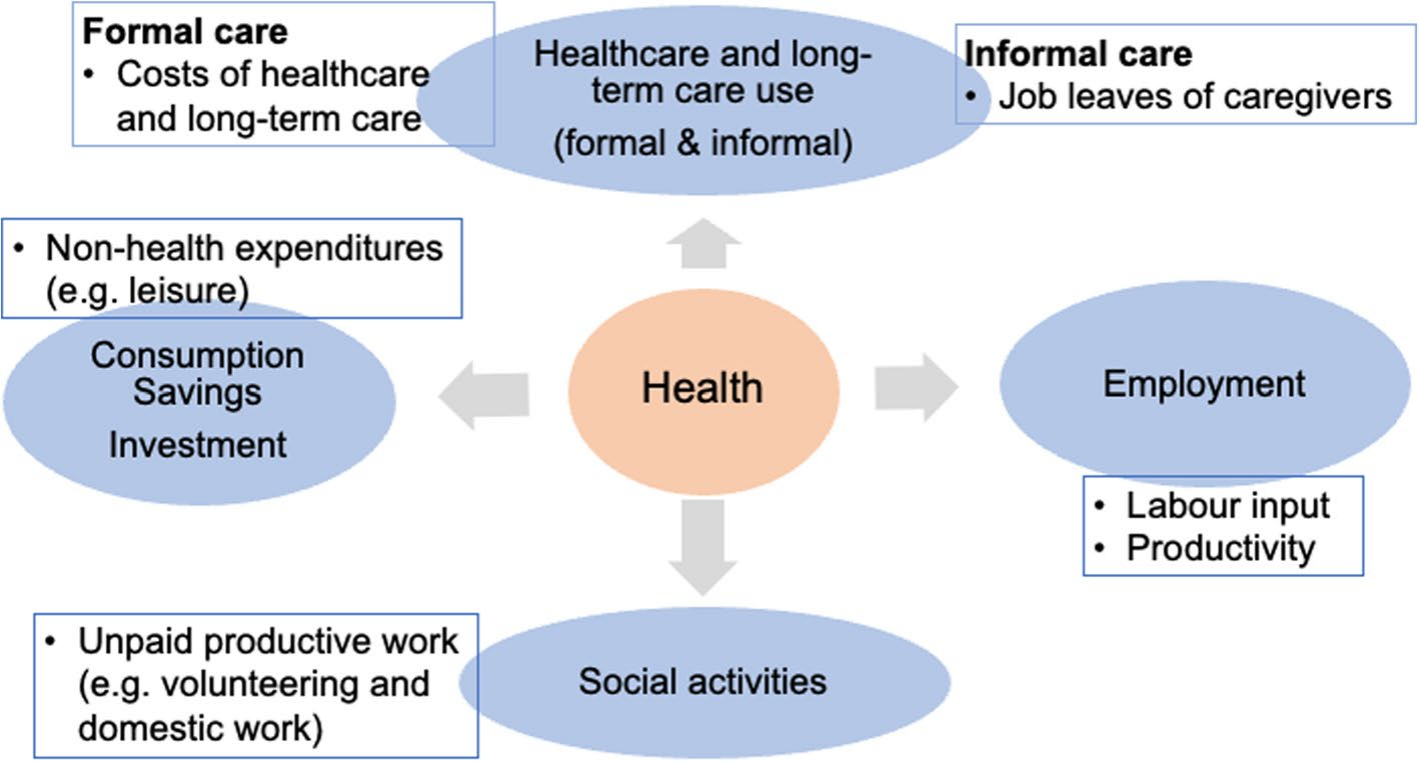
Healthy ageing and social/economic benefits

At a microeconomic level, labour market and financial outcomes (e.g., consumption, saving, and investment) are keys to connecting health with the country’s economy (Fig. 1) [20–23]. Many studies have examined the effects of health on labour market outcomes, such as labour productivity and supply in Western countries [24, 25], suggesting that deteriorating health reduces labour productivity and labour force participation. Among older people, health status works as a *push* factor that pushes them out of the labour market [26].

For financial outcomes, it is plausible that healthier individuals are more active than their unhealthier counterparts in saving for their retirement, expecting that their life expectancy is long, and investing in non-medical consumption, including expenditures on leisure activities [20, 21]. Older adults would be subject to weaker constraints imposed by ill-health in their decision-making in these financial activities by remaining in good health.

The population’s health status can be linked to health expenditures. Growth in social expenditures, including medical and long-term care spending, may be induced by the ageing of populations. In health economics research, population ageing itself may not be regarded as one of the principal drivers of healthcare expenditure [27]; however, its contribution to moderate growth in medical expenditures is still expected [23]. Meanwhile, increasing long-term care costs incurred by population ageing can be significant, even with a low base, and potentially larger when informal care costs are considered [23].

Moreover, older adults, particularly after retirement, play an important role outside the labour market through unpaid productive activities, such as volunteering, childcare, and domestic work. These activities may not be regarded as economic activities; however, the contributions made by the social engagement of older people, which can be determined by their health status, are important. Thus, healthy ageing potentially results in economic benefits beyond mere health benefits. Therefore, this study aims to analyse the association between healthy ageing and various economic indicators, expanding the literature by providing more in-depth data and insights on the linkages than prior studies have done.

## Methods

### Data collection

#### Japan Household Panel Survey (JHPS/KHPS)

To assess the potential economic effects of healthy ageing, we retrieved data from the Japan Household Panel Survey (JHPS/KHPS), which contained information on economic activities. The JHPS/KHPS comprises a nationwide sample of Japanese individuals aged ≥ 20, whose data were obtained using a two-stage stratified random sampling method based on regional classifications and basic resident registers. The survey began in 2004 and was conducted annually by using the same procedure. In this study, we analysed data up to 2019. Information about the survey (e.g., response rates) is provided in greater detail on the project’s website [28]. We restricted our sample to respondents aged 60–95 to provide an analysis of older individuals. Since not all questions were asked in all waves, our data consisted of an unbalanced panel, resulting in approximately 20,000 person-year observations from 3,000 respondents.

#### Official macro-level (aggregated) statistics and data from a previous work﻿

We also utilised data sources published by the Japanese government and a previous study [29] to estimate the economic effects of healthy ageing. The data included statistics on the number of workers, average wages, the population by age, the number of people requiring long-term care, and medical and long-term care costs. The list of statistics is provided in Appendix Table A-1.

### Economic indicators

To measure the economic effects, we analysed three types of economic indicators obtained from the JHPS/KHPS and identified by previous studies [20–23]: labour market outcomes, financial outcomes, and unpaid productive activities.

Labour market outcomes were measured by employment status (i.e., retired or not), labour productivity, and work hours. We operationally defined those not working for pay as retired, excluding individuals with unusual labour force statuses, such as unemployment, temporal job leave, job-seeking, and suspension from work, following the idea that retirement refers to the status of being out of the labour force with no intent to seek employment [30]. Labour productivity was measured as hourly wages for those in paid work. We obtained hours of work as the hours per day that respondents usually worked. To exclude outliers from the analysis, we excluded those with the top (JPY 31,160.9 per hour or higher) and bottom 1% (JPY 213.5 or lower) of wages. As there were self-employed individuals, calculated hourly wages were not necessarily beyond the statutory minimum wages.

Financial outcomes were measured by savings, securities, and consumption. Savings included ordinary deposits, fixed deposits, instalment savings, gold savings, etc., adjusted by the 2015 consumer price index in Japan. Securities included stocks, bonds, mutual funds, etc., adjusted by the 2015 consumer price index in Japan. To address the high number of missing cases for savings and securities, we imputed the data by linear interpolation for each respondent across years. Consumption included expenses in the last month of the surveys, including those for food (including costs for eating out), health (i.e., total health expenditure), culture and recreation, and social relationships. As these variables were obtained as household-level variables, we equalised them by household size, dividing them by the square root of the number of household members. Furthermore, to address the skewed distributions, we transformed savings, securities, and consumption using the inverse hyperbolic sine transformation [31].

Unpaid productive activities were measured by three types of activities: volunteering, domestic work, and childcare (only for respondents who have a child). These were measured in two ways: (1) hours of activities per week; (2) dichotomised variables that take one if respondents were engaged in these activities (i.e., 0 < hours per week of each activity). Hours of activities were transformed using the inverse hyperbolic sine transformation to address the skewness of their distributions.

To assess whether individuals with functional limitations engage in multiple activities or focus on one primary activity, we additionally analysed the relationship between functional limitations and the number of activities out of four that individuals were engaged in, namely, employment, volunteer work, domestic work, and childcare.

### Functional limitations

While there is no consensus on how to measure healthy ageing, a WHO’s report defines healthy ageing as “the process of developing and maintaining the functional ability that enables well-being in older age” [32]. Following frequently used components of healthy (or successful) ageing [33], we operationally measured the intrinsic capacity of respondents by assessing difficulties in performing basic activities of daily living (ADL), instrumental activities of daily living (IADL), and cognitive functioning. In addition to these three components, to determine functional limitations, we also used the status of certification of the need for public long-term care. Under the public long-term care insurance system in Japan, older people (or their family members) in need of long-term care benefits submit an application to their municipality office. After evaluation by the municipality office, individuals are classified into support need-level 1–2 or LTC need-level 1–5 (a higher level indicates more severe conditions), depending on their level of care needs. We considered people to have functional limitations if they were certified at care need levels 2, 3, 4, or 5, where individuals were likely to undergo cognitive decline and/or need partial support from others for their basic and/or instrumental daily activities.

Household surveys, including the JHPS/KHPS, tend to lack objective health information and contain only subjective health status, such as self-rated health. Although some studies have demonstrated the validity of this measure among people without cognitive impairment [34], using subjective health measures without a health modelling approach can cause endogeneity due to measurement error and other potential biases [35]. To avoid potential endogeneity, we imputed information about each respondent’s functional limitation status by utilising estimates from the National Survey of the Japanese Elderly (NSJE). Using common predictors of functional limitation status between surveys, we obtained the functional limitation equation to predict the functional limitation status among the JHPS/KHPS sample as described below.

#### Data: National Survey of the Japanese Elderly

To complement the health indicators lacking in the JHPS/KHPS, we utilised data derived from the NSJE. The NSJE consists of a nationally representative sample of Japanese adults aged 60 or above, extracted from the basic resident registers using a two-stage stratified random sampling method. The survey began in 1987 (Wave 1) with follow-ups of participants every 3–6 years, adding new samples in 1990 (Wave 2), 1996 (Wave 4), 2000 (Wave 5), and 2012 (Wave 8). The latest survey currently available was conducted in 2017 (Wave 9). The project webpage provides more detailed information about the survey [1].

Our analysis only used data obtained from surveys between Wave 6 (2002) and Wave 9 (2017), which fell within a close year range to that of the JHPS/KHPS. The final sample analysed, with the same age range as the JHPS/KHPS (i.e., 60–95), contained 8,456 person-wave observations by 4,148 individuals, comprising an unbalanced panel.

#### Variables to measure functional limitation

As mentioned in Sects. 2 and 3, we defined the functional limitation status based on ADL), IADL, cognitive functioning, and the certified status for the need for pulic long-term care.

Similar to the Katz index [2], ADL was measured using six items: bathing, dressing, feeding, transferring, going out, and toileting. IADL was measured using four items: shopping for personal items, using a telephone, riding the bus or subway alone, and performing light tasks around the house [3]. If a respondent had any difficulties with at least one of the items in either ADL or IADL, they were considered to have functional limitations.

In addition to ADL and IADL, we used cognitive functioning measured by the Short Portable Mental Status Questionnaire [4, 5]. The questionnaire contains nine questions, including the respondent’s home address, interview date, interview day, mother’s maiden name, name of the current prime minister, name of the previous prime minister, a simple calculation, the respondent’s birthday, and age. Respondents who provided four or fewer correct answers out of nine were considered to have moderate cognitive impairment [4] and were defined as having functional limitations.

Furthermore, respondents certified for the need for public long-term care at levels 2, 3, 4, or 5 were also considered to have functional limitations. At these levels, individuals are considered to undergo cognitive decline and/or need partial support from others for their ADL and IADL.

#### Functional limitation prediction

To predict the binary functional limitation status, we formalised its relationship with covariates as:

$$Pr\left({y}_{it}=1|{x}_{it}, {u}_{j}\right)= \frac{exp\left({x}_{it}\beta +{u}_{t}\right)}{1+\text{exp}\left({x}_{it}\beta +{u}_{t}\right)}$$

where ${y}_{it}$ denotes the functional limitation status of a respondent i in a cluster (wave) t, ${x}_{it}$ is a vector of covariates, and ${u}_{t}$ represents the random effects. To build a model to impute functional limitation status from the NSJE into the JHPS/KHPS, it is necessary to use common predictors included in both surveys. Among the demographic, socioeconomic, and behavioural determinants of healthy ageing [6, 7], we used age, age squared, sex, marital status (single or not), the number of household members having at least one child, employment status, house ownership, educational attainment (i.e., high school graduates defined as 12–15 years of education completed and university graduates with 16 + years of education), self-rated health, cigarette smoking, alcohol consumption in addition to controls for years and residential areas by scale (descriptive statistics in Table 1).

**Table 1:** Descriptive statistics: National Survey of the Japanese Elderly

Variables	N	% or mean	SD
Functional limitation	8,540	21.0%	
Female	8,540	56.3%	
Employment status	8,540	20.9%	
Age	8,540	77.50	7.19
Marital status (Single or not)	8,540	40.4%	
Household size	8,540	1.88	1.67
Having a child	8,540	94.1%	
House ownership	8,540	88.9%	
SRH: Very bad	8,540	2.6%	
SRH: Bad	8,540	15.5%	
SRH: Fair	8,540	42.5%	
SRH: Good	8,540	24.8%	
SRH: Very good	8,540	14.5%	
Education: High school	8,540	0.1%	
Education: University or higher	8,540	26.1%	
Cigarette smoking	8,540	6.8%	
Alcohol consumption	8,540	11.0%	

Note: SD represents standard deviation; SRH represents self-rated health

The result of the analysis on the functional limitation status is shown in Table 2, indicating that many of the demographic and health-related variables are useful predictors of functional limitation status. Based on this estimation result, we obtained a linear prediction of the probability of the functional limitation status among the NSJE sample. We defined respondents with a predicted probability larger than 0.5 as having functional limitations; our predictive values were very high (Table 3).

**Table 2:** Results: Functional limitation function

Covariates	beta (SE)	Covariates	beta (SE)
Female	0.32*	SRH: Bad	-2.69**
	(0.14)	(Ref: Very bad)	(0.30)
Age	-0.56**	SRH: Fair	-4.51**
	(0.15)		(0.32)
Age^2^	0.00**	SRH: Good	-5.48**
	(0.00)		(0.35)
Marital status (single or not)	0.30*	SRH: Very good	-6.48**
	(0.12)		(0.40)
Household size	0.15**	SRH: DK	-3.42**
	(0.03)		(1.21)
Having a child	-0.39	Cigarette smoking	0.19
	(0.24)		(0.19)
Employment status	-1.27**	Alcohol consumption	-0.69**
	(0.19)		(0.13)
House ownership	-0.33	Constant	14.54*
	(0.17)		(5.92)
Education: High school	-0.29	Var(_cons[Individuals])	3.57**
	(0.15)		(0.51)
Education: University+	-0.45	Year	Yes
	(0.28)	Area-by-scale	Yes
		Age range	60–95
		Observations	8,456
		Individuals	4,148

Note: Estimated using a multilevel mixed-effects generalised linear model with a binomial distribution of the dependent variable and the logit link function; Weighted by population weights estimated by national statistics of population estimates and censuses based on age, sex, and regions at baseline for samples extracted in 1987, 1999, and 2012; Values represent coefficients, with robust standard errors reported in parentheses; ** *p*<0.01, * *p*<0.05

**Table 3:** Prediction of functional limitation status in the NSJE sample

		Actual functional limitation		
		0	1	Total
Predicted functional limitation	0	6,637	488	6,472
		93.15%	6.85%	100%
	1	112	1,303	1,984
		7.92%	92.08%	100%
	Total	6,749	1,669	8,540

Note: Actual functional limitation is defined as the status where a respondent has any difficulties in ADL or IADL, their cognitive functioning score being less than four (Moderate decline), or is certified for long-term care needs at levels 2–5

Before applying the estimated parameters in the JHPS/KHPS sample, we also estimated the function of the random effects ${u}_{t}$ as a linear function:

$${u}_{t}=c+\delta {x}_{i}+{e}_{i}$$

where c is a constant term, ${x}_{i}$ is a vector of the aforementioned covariates at baseline (or the closest information from the baseline if a variable at baseline is missing) with parameters $\delta$, and ${e}_{i}$ denoting a stochastic disturbance. To account for the prediction error from this linear prediction, we obtained random numbers following the normal distribution with the same mean and standard deviation as the residual, specifying a random-number seed on Stata generated by Microsoft Excel for Mac 2021.

Using these parameters and random numbers, we imputed the functional limitation status in the JHPS/KHPS sample, defining an imputed probability larger than 0.5 as having functional limitations. To test the robustness, we conducted the analysis by using the continuous probability. 

For the sake of the additional robustness test, we estimated the number of functional limitations comprising a total of 19 items from ADL, IADL, and cognitive functioning in the same procedure. We also used this continuous functional limitations score to assess the relationship with the outcomes of our interest. The results are available upon request.

### Empirical strategy

#### Micro-econometric analysis on the linkage between functional limitations and economic indicators

Utilising the panel data structure of the JHPS/KHPS, we expressed the association between health and economic indicators as follows:

$${Y}_{it}={\beta }_{0}+{\beta }_{1}{Functional\ limitations}_{it}+{\beta }_{2}{x}_{it}+{\gamma }_{tl}+{\mu }_{i}+{\epsilon }_{it}$$

where ${Y}_{it}$ denotes the economic indicators of our interest for individual *i* in year t, ${Functional limitations}_{it}$ indicates the imputed functional limitation status, taking the value one if a person has functional limitations. ${x}_{it}$ is a vector of control variables, ${\beta }_{0}$ is a constant term, ${\beta }_{1}$ is a parameter of our interest, ${\beta }_{2}$ are coefficients of covariates, and ${\epsilon }_{it}$ represents a stochastic disturbance.

The control variables include demographic and socioeconomic variables that can be confounders of the associations between functional limitation and economic indicators: age, age squared, eligibility for employees’ pension for the flat benefits and wage-proportional benefits (judged by a respondent’s birth year), marital status (i.e., single or not), having at least one child, the number of household members, house ownership, employment status, contract type (full- or part-time), and equalised household income. We used different controls for each category of dependent variables because confounders of the association between health and labour market outcomes/savings, investment, and consumption/unpaid activities would not be identical. Specifically, when analysing labour market outcomes, pension eligibility affects retirement decision-making [36], so variables indicating pension eligibility were included in the analysis of retirement status. Meanwhile, to analyse productivity and hours of work, we included the contract type because contracts for employed individuals may largely determine wage rates and working hours. Furthermore, as productivity and work hours were observable only among workers, the models for these dependent variables include inverse mills ratios, estimated from an employment status function (Appendix Table A-2), to address sample selection. The analysis of labour market outcomes was restricted to individuals aged 60–89 since no respondent worked beyond this age range. In contrast, to analyse financial outcomes (i.e., savings, investment, and consumption) and unpaid activities, we controlled for retirement status and income, as these inevitably affect time and budget constraints.

Moreover, we controlled for prefecture-by-city-scale-by-year fixed effects (${\gamma }_{tl}$) and individual fixed effects (${\mu }_{i}$) to analyse all outcomes. The city scale includes three categories of ordinance-designated cites, other cities, and towns and villages. Intuitively, prefecture-by-scale-by-year fixed effects control for unobserved factors varying across time and locations (e.g., economic conditions and policy changes). In addition, time-invariant individual heterogeneity, such as personality and genes, is controlled by individual fixed effects. To control for high-dimensional fixed effects, we conducted estimations using a Stata command: *reghdfe* [37].

To partially correct for biases due to non-response, we used two types of weights for all the analyses: cross-sectional and longitudinal. These approaches are similar to multiple imputations grounded in missing at random or ignorable non-response, consistent with previous studies [38, 39]. To mitigate the non-response bias at baseline, we estimated cross-sectional weights by factors (i.e., age, sex, marital status, education, employment status, and residential regions) using the closest census and national surveys conducted by the Japanese government. Moreover, to mitigate biases caused by sample attrition during follow-ups, we calculated weights estimated as the inverse probabilities of responding to each wave by a logit model conditional on factors at baseline, which were the same as those used for cross-sectional weights. Ethical approval for the JHPS/KHPS was not required as the data were publicly available. For the NSJE, all the study procedures were approved by the Institutional Review Board of the Tokyo Metropolitan Institute of Gerontology (R21-008).

#### Economic costs of functional limitations

After detecting economic indicators associated with functional limitations, we estimated the economic costs of functional limitations in Japan (i.e., foregone wages and medical and long-term care costs). We described the approaches later. All analyses were conducted using Stata MP, version 17.0 (StataCorp LLC, College Station, USA).

## Results

Table 4 presents descriptive statistics of the JHPS/KHPS sample. The sample contained people with an average age of 68.25 years. Among them, the proportion of individuals estimated to have functional limitations was 3.6%. Approximately half the respondents were employed during the study period.

**Table 4:** Descriptive statistics: JHPS/KHPS

	With functional limitations		Without functional limitations	
Variables	N (Person-year)	% or mean (SD)	N (Person-year)	% or mean (SD)
Age	20,395	67.99 (5.51)	754	75.36 (6.38)
Women	20,395	50.5%	754	66.0%
Retire	20,395	52.3%	754	93.5%
Self-employment	20,395	17.3%	754	4.1%
Marital status (Single or note)	20,395	20.7%	754	37.4%
House ownership	20,395	88.6%	754	76.9%
Having a child	20,395	60.2%	754	54.8%
Household size (N of household members)	20,395	2.6 (1.28)	754	2.38 (1.32)
Hourly wage (JPY)	7,919	3,228.04 (3,873.23)	28	3,779.53 (4,751.61)
Hours of work (Hours/day)	8,640	4.82 (2.75)	30	4.75 (3.18)
Contract type: Full time	8,640	19.7%	30	23.3%
Contract type: Part time	8,640	37.7%	30	13.3%
Consumption: Total (JPY1,000)	18,738	186.76 (178.51)	654	157.98 (105.14)
Consumption: Food (JPY1,000)	18,980	54.54 (31.08)	668	49.03 (32.46)
Consumption: Health (JPY1,000)	18,507	10.42 (21.01)	643	14.12 (22.37)
Consumption: Entertainment (JPY1,000)	17,903	10.31 (33.62)	612	5.84 (14.69)
Consumption: Social (JPY1,000)	18,373	23.01 (30.08)	635	17.52 (36.56)
Equalised household income (JPY10,000)	18,980	355.01 (306.83)	668	281.15 (275.48)
Equalised household savings (JPY10,000)	18,612	926.6 (1285.03)	656	844.21 (1286.41)
Time use: Volunteer (Hours per week)	10,392	0.39 (1.61)	321	0.06 (0.39)
Time use: Domestic work (Hours per week)	9,992	2.33 (2.48)	311	2.22 (2.47)
Time use: Childcare (Hours per week)	10,449	0.2 (1.45)	322	0.09 (0.56)
Participation: Volunteer	10,392	12.5%	321	3.1%
Participation: Domestic work	9,992	73.5%	311	66.2%
Participation: Childcare	10,449	4.9%	322	3.4%

Note: SD represents standard deviations. Descriptive statistics are calculated by the available case basis. Thus, sample size is not always consistent with the ones used in the analyses

### Labour market outcomes

Table 5 presents the estimated parameters of the linkages between functional limitations, labour market outcomes, savings and investment, consumption, and unpaid productive activities. As shown in the table, having a functional limitation is associated with a 3-percentage-point increase in the probability of retirement (standard error [SE]: 0.01). However, we did not find a significant association between functional limitation and productivity/hours of work. To assess heterogeneity among younger individuals whose employment rates were higher than older people, we restricted the age range between 60 and 69, as the associations may become stronger among this group. We observed identical results qualitatively, finding a larger increase in the probability of retirement due to functional limitation in this group (Appendix Table A-3).

**Table 5:** Functional limitation and economic indicators: Labour market and financial outcomes

	Labour market outcome			Savings and investment		Consumption				
	Retirement	Productivity (Hourly wage)	Hours of work	Savings	Securities	Food	Health	Culture and entertainment	Social relationships	Total
Functional limitation	0.03**	-0.11	-0.24	-0.03	-0.04	-0.03	0.29**	-0.01	0.10	-0.01
	(0.01)	(0.16)	(0.21)	(0.11)	(0.09)	(0.04)	(0.07)	(0.07)	(0.08)	(0.03)
Age range	60–89	60–89	60–89	60–95	60–95	60–95	60–95	60–95	60–95	60–95
Observations	21,149	7,853	8,670	19,609	19,874	19,648	19,142	18,466	18,991	19,401
Individuals	3,113	1,555	1,639	2,945	2,976	2,968	2,943	2,889	2,930	2,948
	Time (Hours per week)			Engagement (Yes/No)			Number of activities (Paid and unpaid activities)			
	Volunteer	Domestic work	Childcare	Volunteer	Domestic work	Childcare				
Functional limitation	-0.02	-0.16**	-0.00	-0.02	-0.07*	0.01	-0.13**	-0.10		
	(0.01)	(0.06)	(0.03)	(0.01)	(0.03)	(0.02)	(0.04)	(0.14)		
Age range	60–95	60–95	60–95	60–95	60–95	60–95	60–89	60–69		
Observations	18,746	18,362	10,771	18,746	18,362	10,771	10,771	6,706		
Individuals	2,923	2,848	2,110	2,923	2,848	2,110	2,113	1,525		

Note: Estimated by a linear probability model for a binary variable and linear model for continuous variables; Productivity and hours of work are log-transformed; Savings, securities, consumption, and times for unpaid activities are transformed by the inverse hyperbolic sine transformation; The number of activities is a total count of activities that individuals are engaged in, which includes employment; Values represent coefficients with individual-level cluster robust standard errors in parentheses; For labour market outcomes, models include controls for age, age squared, employees’ pension eligibility for the flat benefits and the wage-proportional benefits (only for retirement), self-employment, contract type (only for productivity and hours of work), marital status, house ownership, having a child, household size, prefecture-by-scale-by-year-fixed-effects, and individual-fixed-effects; The analyses for productivity and hours of work include an additional control of the inverse mills ratios predicted in Appendix Table B-2; For financial outcomes and unpaid activities, models include controls for age, age squared, employment status, marital status, house ownership, having a child, household size, equalised household income transformed by the inverse hyperbolic sine transformation, prefecture-by-scale-by-year-fixed-effects, and individual-fixed-effects; Weighted by cross-sectional and longitudinal weights; ** *p*<0.01, * *p*<0.05

### Savings, investment, and consumption

As for savings, investment, and consumption, we found that functional limitation was only associated with increased health expenditure ($\beta$: 0.29, SE: 0.07).

### Unpaid productive activities

Regarding unpaid activities, we found that individuals with functional limitations were less likely to engage in domestic work. This was measured by both time ($\beta$: -0.16, SE: 0.06) and engagement ($\beta$: -0.07, SE: 0.03). We also found that those with functional limitations tended to engage in a smaller number of paid or unpaid activities ($\beta$: -0.13, SE: 0.04).

For all the economic indicators, instead of using a binary variable to indicate functional limitations, we conducted additional analyses using the continuous probability of having functional limitations and the number of functional limitations. As shown in Appendix Table A-4 and A-5, similar results to those obtained with the binary indicator were obtained when using the continuous probability. Moreover, as for the number of functional limitations, we observed clearer relationships between functional limitations and economic indicators and unpaid productive activities: those with a larger number of functional limitations tended to retire, less invest in securities, and be inactive in volunteer and domestic works (Appendix Table A-6 and A-7).

### Foregone wages

**Fig. 2:**
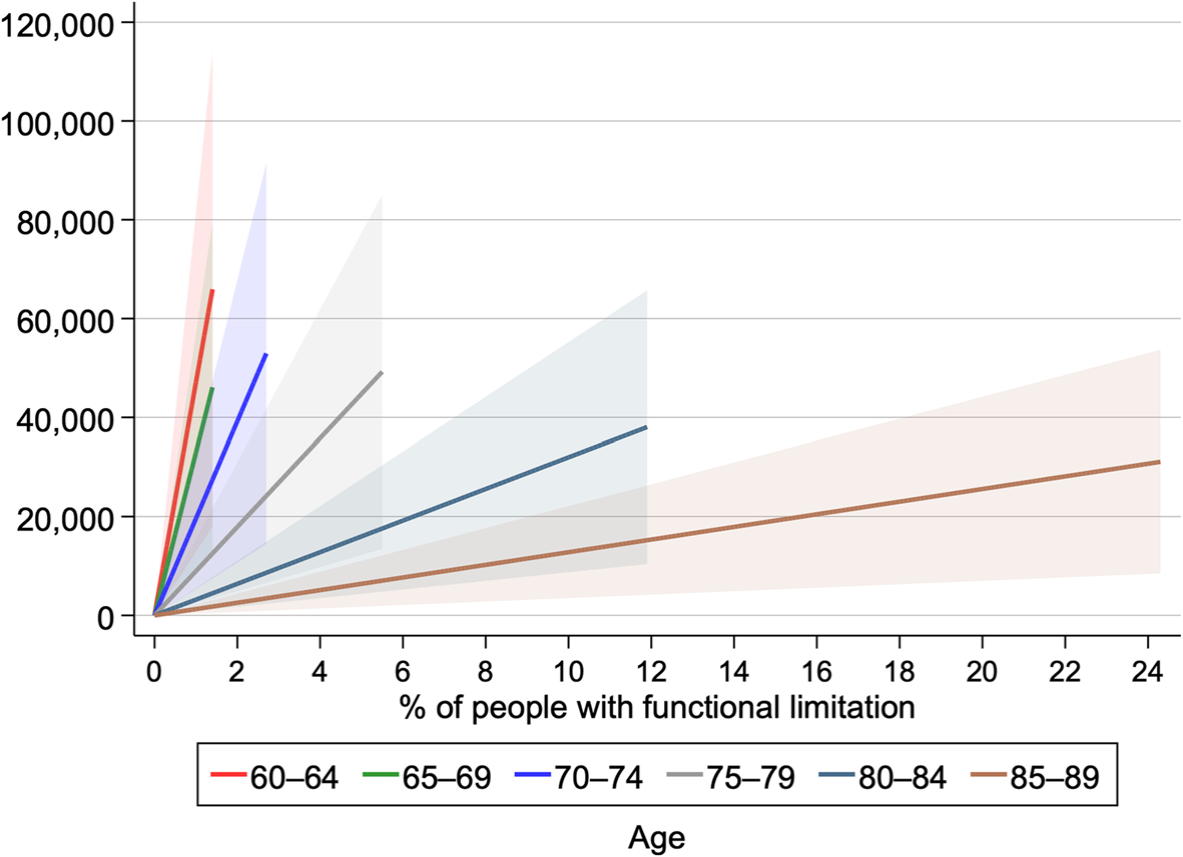
Estimated foregone wages due to functional limitations (Unit of currency: USD 1,000). Note: % of people with functional limitation denotes the proportion of the population with certification of levels 2–5 long-term care needs; Based on the estimate in Table 5, lines represent foregone wages calculated by the point estimate for the probability of retirement due to functional limitations, with the shaded area representing 95% confidence intervals

From the estimates on the labour market outcome, we estimated foregone wages of labour market exits due to functional limitations, which could have been saved by achieving healthy ageing. In this study, annual forgone wages were estimated as follows:

$$Forgone\;wages=\Sigma\left[Pr(retirement\;of\;individuals\;with\;functional\;limitation)\right]\;\ast\;\left[\%\;of\;individuals\;with\;functional\;limitation_j\right]\;\ast\;\left[N\;of\;workers_j\right]\;\ast\;\left[Yearly\;wage_j\right]$$

where j denotes six age ranges: 60–64, 65–69, 70–74, 75–79, 80–84, and 85–89. Although the incidence rate of functional limitation among workers should ideally be used, we could not do so due to data unavailability. Each parameter was obtained from the estimates in this study and official statistics of the Japanese government (Appendix Table A- 8). Our estimates suggest that functional limitation generates an annual economic loss of approximately JPY 32.3 billion ($\approx$ USD 266.4 million; USD 1 is calculated as JPY 120), with 95% confidence intervals ranging from JPY 8.8 billion ($\approx$USD 72.8 million) to JPY 55.9 billion ($\approx$USD 460.1 million). As younger age groups had higher employment rates, the economic losses were larger in these groups (Fig. 2). Particularly, most economic losses were driven by individuals in their 60s (Appendix Figure A- 1).

We further calculated the lifetime foregone wages among those aged between 60 and 68 as follows, considering that the average effective age of labour force exit in Japan is around 68 years old for men [40]. The assumptions made in Appendix Table A-8 about average yearly wages, employment rates, and functional limitation rates were kept the same. As the detailed statistics by each age were not available, it was assumed that these values linearly changed from 60 to 68 years based on available statistics. The estimated retirement probability of people with functional limitation was 0.08, which was obtained in the relevant estimate in Appendix A-3. It was also assumed that individuals with functional limitations would not return to a job post-retirement, and retirement occurred at the beginning of the year. By this calculation, the lifetime forgone wage among those aged 60–68 was estimated to be about JPY 175.6 billion ($\approx$ USD 1.5 billion).

### Medical and long-term care costs

We found that functional limitation was associated with an increase in total health expenditure. The total health expenditure in the JHPS/KHPS was obtained as costs at a household level, and the information about long-term care costs was not available. Therefore, the parameter from our analysis may not reflect the actual costs incurred. With the official statistics and an estimate by a previous study [29], we estimated costs for formal medical and long-term care used by people with functional limitation (i.e., certified for long-term care needs at 2–5 levels). The additional total costs for formal medical and long-term care generated by functional limitation were estimated as follows:

$$Medical\;and\;long-term\;care\;\cos ts\;=\;\Sigma\left[\left(Per-capita\;medical\;and\;long-term\;care\;\cos ts\;of\;those\;with\;functional\;limitation_k\right)-\left(Per-capita\;medical\;and\;long-term\;care\;\cos ts\;of\;those\;with\;no\;functional\;limitation_k\right)\right]\;\ast\;\left[\%\;of\;individuals\;with\;functional\;limitation_k\right]\;\ast\;\left[N\;of\;population_k\right]$$

where k denotes six age categories of 60–64, 65–69, 70–74, 75–79, 80–84, 85–89, and 90 or over. As per-capita medical care costs for individuals with functional limitation by age groups were not available, we used values from a previous study in Japan [29]. Parameters used for the estimate are presented in Appendix Table A-9.

**Fig. 3:**
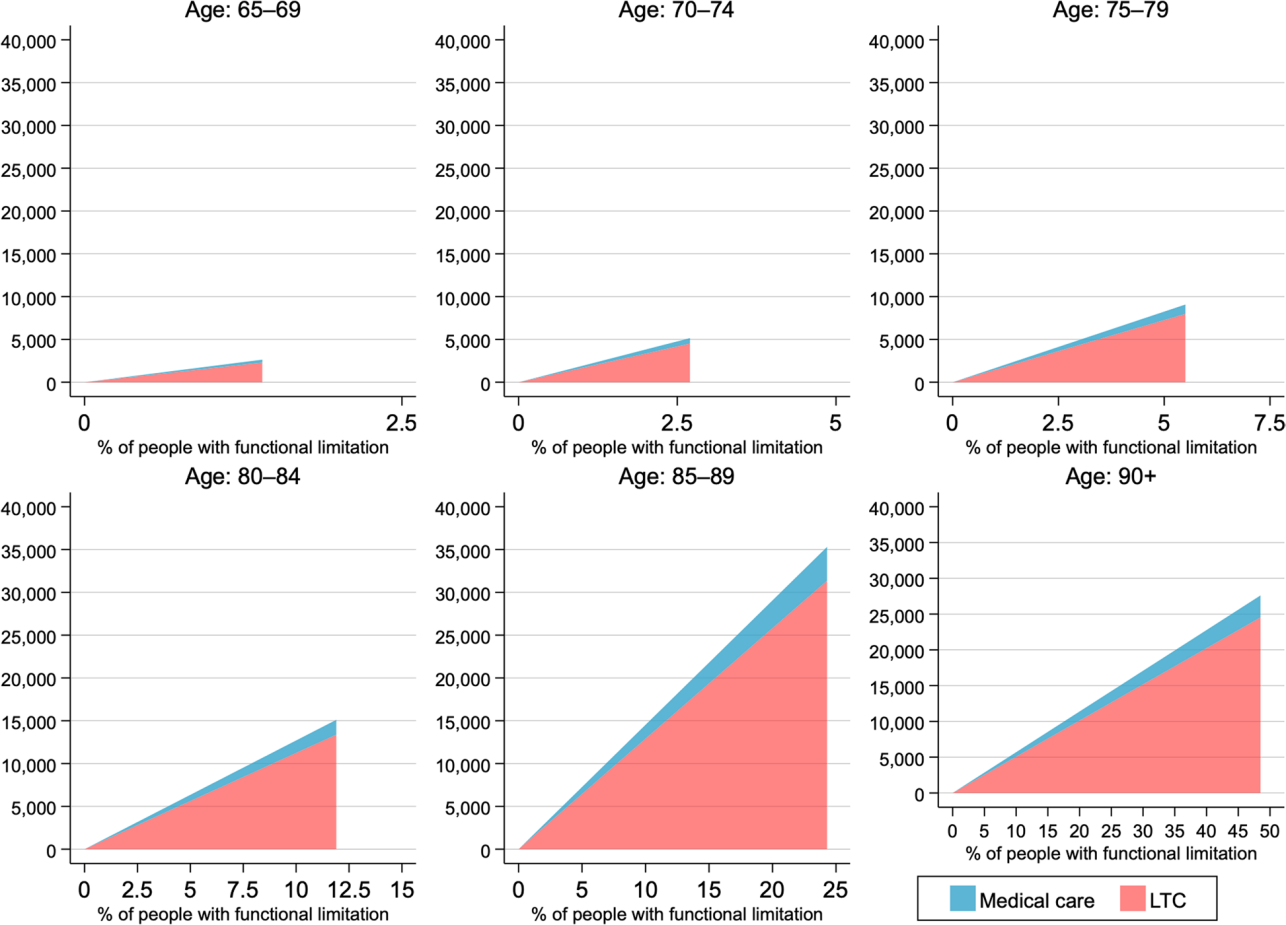
Medical and long-term care costs, additionally generated by functional limitations (Unit of currency: USD 1 million). Note: % of people with functional limitation denotes the proportion of the population with certification of levels 2–5 long-term care needs

Figure 3 and Appendix Table A-10 show the estimated formal medical and long-term care costs, additionally generated by functional limitations, that can be saved if healthy ageing is achieved. In contrast to foregone wages, long-term care costs, rather than medical care costs for older people aged ≥ 85, accounted for a large part of the total costs due to functional limitation. The total additional medical and long-term care costs generated by functional limitations are approximately JPY 8.8 trillion ($\approx$ USD 72.7 billion).

## Discussion

This study aimed to evaluate the linkage between healthy ageing and economic indicators to determine the potential economic benefits of healthy ageing. We found that functional limitation, used as an indicator for healthy ageing, was associated with a higher likelihood of retirement, a higher level of total health expenditures, and a lower probability of engaging in unpaid productive work. We estimated foregone wages and additional medical and long-term care costs, which could be saved by achieving healthy ageing. Our estimates indicate that foregone wages and additional medical/long-term care costs were approximately JPY 32.3 billion ($\approx$ USD 266.4 million) and JPY 8.8 trillion ($\approx$ USD 72.7 billion), respectively. Although it is difficult to contrast these values with estimates from previous studies [10, 11] due to the differences in measurements of economic gains, our findings are qualitatively consistent with them in that healthy ageing can generate economic gains.

In line with existing findings [24–26], poor health was associated with retirement in our study, working as a push factor from the labour market. However, our estimates did not suggest that functional limitation was linked to productivity and work hours among workers, potentially because one’s later-life health status can largely affect whether they work or not, rather than variabilities in productivity among workers. Averting the retirement of people with functional limitations, particularly of the working-age population, is essential not only for the health of the population but for the country’s economy. Furthermore, we found that functional limitation was linked to total health expenditure, indicating that those with functional limitations spent more on health expenditure.

In our further estimates of additional medical and long-term care costs, we found that long-term care costs, rather than medical care costs, were a significant driver of incurred costs. This is likely because people may use healthcare services even if they do not have functional limitations. While we did not provide an economic value for volunteer and domestic works, engaging in these activities generates non-monetary values and may allow older people to live independent lives without requiring intensive long-term care, which is well in line with our estimates that long-term care costs are largely saved by healthy ageing.

In this study, we did not cover crucial indicators. For instance, due to data unavailability, we could not estimate the costs of informal care that fall on individuals other than those with functional limitations. Family members of people with functional limitations usually bear the burdens of caregiving, compelling them to reduce their working hours or quit their job. A previous study identified 99,100 annual job leaves due to caregiving in Japan, with 64.3% remaining out of the labour market even two years after their leaves [41]. The study estimated annual foregone wages induced by this to be JPY 182.1 billion ($\approx$ USD 1.4 billion), which is higher than our estimates of the foregone wages of people with functional limitations themselves. In addition to the financial costs of informal care, gender inequality is also a concern. In many countries, women tend to bear the burden of caregiving more than men [42]. Consequently, they may be required to refrain from participating in social and economic activities. Also, we could not include costs generated by premature death since the JHPS/KHPS did not collect this information. Therefore, the actual economic benefits that can be produced by achieving healthy ageing may be much larger than our estimates.

### Potential interventions to achieve healthy ageing

To achieve healthy ageing and reduce the burden of disease, clinical and behavioural interventions, as well as intersectoral policies, are necessary [5]. Previous studies suggest that modifiable factors, including smoking, alcohol consumption, physical activity, and abnormal bodyweight, account for healthy ageing [43, 44]. Therefore, addressing these behavioural determinants of health, which requires a health-in-all-policies approach, is essential to promote progress towards healthy ageing. Furthermore, the life course approach must be recognised as the health status of older adults is not only determined by current determinants but also by past ones [45]. This indicates the need for interventions for children, adolescents, adults, and older adults to achieve healthy ageing [46].

Interventions can still be effective in preventing and delaying functional limitations, even for older adults. Existing studies suggest that interventions targeting modifiable factors, such as social interactions, smoking, physical activity, nutritional status, cognitive training, and occupational and physical therapy sessions with home modifications, are effective [47–49]. To provide effective interventions for older adults, multidisciplinary and multifactorial interventions with individualised programmes, case management, long-term follow-up, physical exercise component, and technology can be key to success [46, 50, 51]. Moreover, some studies suggest that health promotion among older adults is cost-effective [52–54]. Although we solely evaluated the potential effects of healthy ageing, investment in healthy ageing can be cost-effective by improving the health of older people, generating additional economic and financial returns.

### Policy implications

Our estimates point to three policy and research concerns that policymakers and researchers should understand: labour policies (e.g., retirement policy and occupational safety), employment opportunities in the health and long-term care sectors, and data collection for healthy ageing.

First, there is a need for labour policies that incentivise older people to work. Despite good health and willingness to work, retirement policies may discourage older people from being employed, such as early pension/retirement age and a large wage decline after reaching a certain age. Work environments, including hours/days of work and occupational safety, are important so that older people can work healthily, even if their physical or cognitive functioning mildly declines compared to their younger ages.

Second, the reduction of healthcare and long-term care costs should not be automatically regarded as ‘benefits’, as they create employment opportunities in the health and long-term care or other sectors, contributing to the country’s economy. However, a shortage of labour force and financial sustainability of public health and long-term care systems is common in many countries. Thus, the reduction of financial costs and burdens borne by workers through health improvement may mitigate these challenges.

Third, data collection and analysis are necessary to better understand and evaluate the economic benefits of healthy ageing in each country. Our framework highlights the need to collect data on health indicators (e.g., ADL, IADL, and cognitive functioning), labour market outcomes (e.g., employment status, days/hours of work, and wage), expenditures (e.g., health and long-term care spending and leisure expenditures), financial activities (e.g., savings, consumption, and investment), and social activities (e.g., volunteer, domestic work, and group activities) to properly assess the benefits of healthy ageing. Depending on the country’s context, such as size, socio-economic development, or cultural context, policymakers should tailor their policies to reflect the social or economic values of the country and the sections involved in necessary policies for achieving healthy ageing.

### Limitations

This study has several limitations. First, the sample of a household survey may not be representative of the general population, even though we attempted to address this issue by using cross-sectional and longitudinal weights. People willing to respond to complex questions (e.g., financial assets) may be biased (e.g., healthier and wealthier than the average population). As self-reported measures have a subjective element, responses to complex questions, including one’s wage, may not always be accurate. Therefore, future research should consider obtaining data from public records (e.g., tax and medical and long-term care records) in combination with survey data from people of various ages, especially older people.

Second, the JHPS/KHPS does not have a large sample of older individuals as it focuses on education and employment, primarily collecting data from younger individuals. Although we imputed the functional limitation status in the JHPS/KHPS using data from the NSJE, which seemed to work well, the different age distribution between the surveys (i.e., NSJE contained more older respondents than JHPS/KHPS) could lead to imprecise estimates if the determinants of functional limitation varied significantly across age groups in the samples.

Third, the JHPS/KHPS does not collect information about deaths. By further analysing foregone wages due to premature death, more accurate estimates could be feasible.

Fourth, our estimate of additional medical care costs due to functional limitations used data from one city in Japan, as detailed information about medical care costs by long-term care need level was not available. Although we found that long-term care costs were a significant driver of additional costs rather than medical care costs, relying on medical care costs data from a specific city may lead to discrepant estimates from genuine costs. Disparities in healthcare resources across regions can impact medical care costs. Therefore, future studies should consider nationwide data linked to public records.

Fifth, the definition of healthy ageing in this study was solely based on one’s health status (i.e., functional limitation), while excluding other factors that contribute to healthy ageing, such as equity, cohesion, and well-being [55]. If a broader concept of healthy ageing is adopted, its benefits may also expand.

Sixth, while we partly addressed the issue of endogeneity by avoiding self-reported measures and adopting a high-dimensional fixed-effect model, the linkages between health and economic indicators can still be endogenous due to factors such as reverse causality and unobserved time-varying confounding. Therefore, better evidence using causal inference is required.

By analysing data from Japan, we suggest that achieving healthy ageing can produce economic benefits by preventing exits from the labour market due to health issues and reducing medical and long-term care costs. Also, healthy older people can make contributions through nonpaid productive activities. Policymakers and researchers should understand the linkages between healthy ageing and the economy to address a wide range of health determinants through multisectoral involvement from both health and non-health sectors to provide cost-effective interventions for older people.

## Supplementary Information

**Additional file 1: Appendix Table A-1.** List of the data sources. **Appendix Table A-2.** Predictors of employment status. **Appendix Table A-3.** Functional limitation and economic indicators: Labour market outcomes among people aged 60-69. **Appendix Table A-4.** Continuous probability of having functional limitations and economic indicators: Labour market and financial outcomes. **Appendix Table A-5.** Continuous probability of having functional limitations and economic indicators: Unpaid activities. **Appendix Table A-6.** Number of functional limitations and economic indicators: Labour market and financial outcomes. **Appendix Table A-7.** Number of having functional limitations and economic indicators: Unpaid activities and number of activities. **Appendix Table A-8.** Parameters and estimates of foregone wages due to functional limitation. **Appendix Table A-9.** Per-capita annual costs for health and long-term care. **Appendix Table A-10.** Additional medical and long-term care costs. **Appendix Figure A-1.** Estimated foregone wages due to functional limitation among people in their 60s.

## Data Availability

The authors do not have the right to share the data.
