# Supplementary material for: Economic effects of healthy ageing: functional limitation, forgone wages, and medical and long-term care costs
Source: Health Econ Rev. 2023 May 10;13:28. doi: 10.1186/s13561-023-00442-x (PMC10170784; doi:10.1186/s13561-023-00442-x)
Supplement: Supplementary file 1 — Additional file 1: Appendix Table A-1. List of the data sources. Appendix Table A-2. Predictors of employment status. Appendix Table A-3. Functional limitation and economic indicators: Labour market outcomes among people aged 60-69. Appendix Table A-4. Continuous probability of having functional limitations and economic indicators: Labour market and financial outcomes. Appendix Table A-5. Continuous probability of having functional limitations and economic indicators: Unpaid activities. Appendix Table A-6. Number of functional limitations and economic indicators: Labour market and financial outcomes. Appendix Table A-7. Number of having functional limitations and economic indicators: Unpaid activities and number of activities. Appendix Table A-8. Parameters and estimates of foregone wages due to functional limitation. Appendix Table A-9. Per-capita annual costs for health and long-term care. Appendix Table A-10. Additional medical and long-term care costs. Appendix Figure A-1. Estimated foregone wages due to functional limitation among people in their 60s. [file 13561_2023_442_MOESM1_ESM.docx]

Supplementary material for

**Economic effects of healthy ageing:**

**Functional limitation, forgone wages, and medical and long-term care costs**

**Contents**

**Appendix A: Appendix Tables and Figure**

- Appendix Table A-1. List of the data sources
- Appendix Table A-2. Predictors of employment status
- Appendix Table A-3. Functional limitation and economic indicators: Labour market outcomes among people aged 60-69
- Appendix Table A-4. Continuous probability of having functional limitations and economic indicators: Labour market and financial outcomes
- Appendix Table A-5. Continuous probability of having functional limitations and economic indicators: Unpaid activities
- Appendix Table A-6. Number of functional limitations and economic indicators: Labour market and financial outcomes
- Appendix Table A-7. Number of having functional limitations and economic indicators: Unpaid activities and number of activities
- Appendix Table A-8. Parameters and estimates of foregone wages due to functional limitation
- Appendix Table A-9. Per-capita annual costs for health and long-term care
- Appendix Table A-10. Additional medical and long-term care costs
- Appendix Figure A-1. Estimated foregone wages due to functional limitation among people in their 60s

**Appendix A: Appendix Tables and Figures**

Appendix Table A-1. List of the data sources

| Publisher | Statistics name | Information used | Year | URL |
| --- | --- | --- | --- | --- |
| Ministry of Internal Affairs and Communications (Statistics Bureau of Japan) | Population Estimates | The number of the population | 2019 | <https://www.stat.go.jp/english/data/jinsui/index.html> |
| Ministry of Health, Labour, and Welfare | Basic Survey on Wage Structure | Per-year average wages of workers | 2019 | <https://www.mhlw.go.jp/english/database/db-l/wage-structure.html> |
| Ministry of Internal Affairs and Communications (Statistics Bureau of Japan) | Labour Force Survey | Employment rates | 2019 | <https://www.stat.go.jp/english/data/roudou/index.htm> |
| Ministry of Health, Labour, and Welfare | Report on Long-Term Care Insurance | The number of the population certified for long-term care need | 2019 | <https://www.mhlw.go.jp/toukei/list/84-1.html> |
| Ministry of Health, Labour, and Welfare | Statistics of Long-term Care Benefit Expenditures | Long-term care costs | 2019 | <https://www.mhlw.go.jp/toukei/list/45-1.html> |
| Tomata et al. (2014) | Osaki city, Miyagi, Japan | Medical care costs of people aged 65+ certified for long-term care need | 2007 | <https://doi.org/10.11236/jph.61.11_679> |

Appendix Table A-2. Predictors of employment status

| Covariates | beta (SE) |
| --- | --- |
| Female | -1.89** |
|  | (0.18) |
| Age | -0.43* |
|  | (0.18) |
| Age^2^ | 0.00 |
|  | (0.00) |
| Pension eligibility: Flat benefits | -0.07 |
|  | (0.22) |
| Pension eligibility: Wage-proportional beneﬁts | -0.15 |
|  | (0.10) |
| Marital status (Single or not) | 0.27 |
|  | (0.17) |
| House ownership | -0.22 |
|  | (0.17) |
| Having a child | 0.16* |
|  | (0.06) |
| Household size | 0.04 |
|  | (0.04) |
| Education: High school | -0.22 |
|  | (0.18) |
| Education: College | -0.30 |
|  | (0.33) |
| Education: University+ | -0.39 |
|  | (0.23) |
| Year | Yes |
| Prefecture-by-scale | Yes |
| Constant | 24.77** |
|  | (6.02) |
| Age range | 60-89 |
| Observations | 25,830 |
| Individuals | 3,984 |

Note: Estimated by a random-effects probit model; The dependent variable is a dichotomised employment status, taking one if a respondent is in paid work at the time of the surveys; Values represent coefficients with individual-level cluster robust standard errors.

Appendix Table A-3. Functional limitation and economic indicators: Labour market outcomes among people aged 60–69

|  | Labour market outcomes | | |
| --- | --- | --- | --- |
|  | Retirement | Productivity  (Hourly wage) | Hours of work |
| Functional limitation | 0.08** | -0.42 | 0.15 |
|  | (0.03) | (0.24) | (0.18) |
| Age range | 60–69 | 60–69 | 60–69 |
| Observations | 12,910 | 6,271 | 6,853 |
| Individuals | 2,509 | 1,407 | 1,481 |

Note: Estimated using a linear probability model for retirement and a linear model for productivity and hours of work; Productivity and hours of work are log-transformed; Values represent coefficients with individual-level cluster-robust standard errors in parentheses; Controlled for age, age squared, employee's pension eligibility for the flat benefits and wage-proportional benefits (only for retirement), self-employment, contract type (only for productivity and hours of work), marital status, house ownership, having a child, household size, prefecture-by-scale-by-year-fixed-effects, and individual-fixed-effects; The analyses for productivity and hours of work include an additional control of the inverse mills ratios predicted in Appendix Table A-2; Weighted by cross-sectional and longitudinal weights.

Appendix Table A-4. Continuous probability of having functional limitations and economic indicators: Labour market and financial outcomes

|  | Labour market outcomes | | | Savings and investment | | Consumption | | | | |
| --- | --- | --- | --- | --- | --- | --- | --- | --- | --- | --- |
|  | Retirement | Productivity  (Hourly wage) | Hours of work | Savings | Securities | Total | Food | Health | Culture and entertainment | Social relationships |
| ln(Pr(Functional limitation)) | 0.07** | 0.02 | -0.02 | -0.02 | -0.49 | 0.00 | -0.00 | 0.09** | -0.02 | 0.00 |
|  | (0.00) | (0.01) | (0.01) | (0.02) | (0.28) | (0.01) | (0.01) | (0.01) | (0.02) | (0.02) |
| Age range | 60–89 | 60–89 | 60–89 | 60–95 | 60–95 | 60–95 | 60–95 | 60–95 | 60–95 | 60–95 |
| Observations | 21,149 | 7,853 | 8,681 | 19,713 | 19,874 | 19,758 | 19,257 | 18,581 | 19,104 | 19,514 |
| Individuals | 3,113 | 1,555 | 1,640 | 2,952 | 2,976 | 2,975 | 2,950 | 2,895 | 2,937 | 2,954 |

Note: Estimated using a linear probability model for a binary outcome and a linear model for continuous outcomes; Productivity and hours of work are log-transformed; Savings, securities, and consumption are transformed using the inverse hyperbolic sine transformation; Values represent coefficients with individual-level cluster-robust standard errors in parentheses; the models for labour market outcomes include controls for age, age squared, employees' pension eligibility for the flat benefits and wage-proportional benefits (only for retirement), self-employment, contract type (only for productivity and hours of work), marital status, house ownership, having a child, household size, prefecture-by-scale-by-year-fixed-effects, individual-fixed-effects, and the inverse mills ratios predicted in Appendix Table A-2; the models for financial outcomes include controls for age, age squared, employment status, marital status, house ownership, having a child, household size, equalised household income transformed using the inverse hyperbolic sine transformation, prefecture-by-scale-by-year-fixed-effects, and individual-fixed-effects; Weighted by cross-sectional and longitudinal weights.

Appendix Table A-5. Continuous probability of having functional limitations and economic indicators: Unpaid activities

|  | Time  (Hours per week) | | | Engagement  (Yes/No) | | | Number of activities  (Paid and unpaid activities) | |
| --- | --- | --- | --- | --- | --- | --- | --- | --- |
|  | Volunteer | Domestic work | Childcare | Volunteer | Domestic work | Childcare |  |  |
| ln(Pr(Functional limitation)) | -0.01* | -0.01 | 0.00 | -0.01** | -0.01* | 0.00 | -0.08** | -0.09** |
|  | (0.00) | (0.01) | (0.00) | (0.00) | (0.00) | (0.00) | (0.01) | (0.01) |
| Age range | 60–95 | 60–95 | 60–95 | 60–95 | 60–95 | 60–95 | 60–90 | 60–69 |
| Observations | 18,859 | 18,472 | 10,905 | 18,859 | 18,472 | 10,905 | 10,771 | 6,706 |
| Individuals | 2,933 | 2,857 | 2,119 | 2,933 | 2,857 | 2,119 | 2,113 | 1,525 |

Note: Estimated by a linear model for time and linear probability model for engagement; Time for unpaid activities is transformed by the inverse hyperbolic sine transformation; The number of activities is a total count of activities that individuals are engaged in, which includes employment, volunteer work, domestic work, and childcare; Values represent coefficients with individual-level cluster robust standard errors in parentheses; Controlled for age, age squared, employment status, marital status, house ownership, having a child, household size, equalised household income transformed by the inverse hyperbolic sine transformation, prefecture-by-scale-by-year-fixed-effects, and individual-fixed-effects; Weighted by cross-sectional and longitudinal weights.

Appendix Table A-6. Number of functional limitations and economic indicators: Labour market and financial outcomes

|  | Labour market outcomes | | | Savings and investment | | Consumption | | | | |
| --- | --- | --- | --- | --- | --- | --- | --- | --- | --- | --- |
|  | Retirement | Productivity  (Hourly wage) | Hours of work | Savings | Securities | Total | Food | Health | Culture and entertainment | Social relationships |
| ln(Functional limitation) | 0.11** | 0.11 | -0.15 | -0.02 | -0.14* | 0.00 | -0.02 | 0.31** | -0.03 | -0.05 |
|  | (0.01) | (0.08) | (0.08) | (0.02) | (0.07) | (0.02) | (0.03) | (0.06) | (0.07) | (0.07) |
| Age range | 60–89 | 60–89 | 60–89 | 60–95 | 60–95 | 60–95 | 60–95 | 60–95 | 60–95 | 60–95 |
| Observations | 21,162 | 7,866 | 8,670 | 19,713 | 19,874 | 19,482 | 19,731 | 19,220 | 18,538 | 19,071 |
| Individuals | 3,112 | 1,557 | 1,639 | 2,952 | 2,976 | 2,949 | 2,969 | 2,945 | 2,891 | 2,932 |

Note: Estimated by a linear probability model for a binary outcome and linear model for continuous outcomes; The number of functional limitations is transformed by the inverse hyperbolic sine transformation; Productivity and hours of work are log-transformed; Savings, securities, and consumption are transformed by the inverse hyperbolic sine transformation; Values represent coefficients with individual-level cluster robust standard errors in parentheses; For labour market outcomes, models include controls for age, age squared, employees' pension eligibility for the flat benefits and the wage-proportional beneﬁts (only for retirement), self-employment, contract type (only for productivity and hours of work), marital status, house ownership, having a child, household size, prefecture-by-scale-by-year-fixed-effects, individual-fixed-effects, and the inverse mills ratios predicted in Appendix Table A-2; For financial outcomes, models include controls for age, age squared, employment status, marital status, house ownership, having a child, household size, equalised household income transformed by the inverse hyperbolic sine transformation, prefecture-by-scale-by-year-fixed-effects, and individual-fixed-effects; Weighted by cross-sectional and longitudinal weights.

Appendix Table A-7. Number of functional limitations and economic indicators: Unpaid activities and number of activities

|  | Time  (Hours per week) | | | Engagement  (Yes/No) | | | Number of activities  (Paid and unpaid activities) | |
| --- | --- | --- | --- | --- | --- | --- | --- | --- |
|  | Volunteer | Domestic work | Childcare | Volunteer | Domestic work | Childcare |  |  |
| ln(Functional limitation) | -0.04** | -0.15** | -0.01 | -0.03** | -0.08** | 0.01 | -0.19** | -0.16* |
|  | (0.01) | (0.04) | (0.02) | (0.01) | (0.02) | (0.01) | (0.04) | (0.06) |
| Age range | 60–95 | 60–95 | 60–95 | 60–95 | 60–95 | 60–95 | 60–89 | 60–69 |
| Observations | 18,825 | 18,447 | 10,832 | 18,825 | 18,447 | 10,832 | 10,771 | 6,706 |
| Individuals | 2,926 | 2,850 | 2,115 | 2,926 | 2,850 | 2,115 | 2,113 | 1,525 |

Note: Estimated by a linear model for time and linear probability model for engagement; The number of functional limitations is transformed by the inverse hyperbolic sine transformation; The number of activities is a total count of activities that individuals are engaged in, which includes employment, volunteer work, domestic work, and childcare; Times for unpaid activities are transformed by the inverse hyperbolic sine transformation; Values represent coefficients with individual-level cluster robust standard errors in parentheses; Controlled for age, age squared, employment status, marital status, house ownership, having a child, household size, equalised household income transformed by the inverse hyperbolic sine transformation, prefecture-by-scale-by-year-fixed-effects, and individual-fixed-effects; Weighted by cross-sectional and longitudinal weights.

Appendix Table A-8. Parameters and estimates of foregone wages due to functional limitation (Unit of currency: JPY)

|  | Average yearly wage | Employment rate | | Total  Population | Functional limitation rate | Estimated retirement probability of people with functional limitation | Estimated retirement probability of people with functional limitation (Lower) | Estimated retirement probability of people with functional limitation (Upper) |
| --- | --- | --- | --- | --- | --- | --- | --- | --- |
| 60–64 | 3,465,800 | 0.70441 | | 7,524,000 | 0.01379019 | 0.0307630 | 0.0084062 | 0.0531197 |
| 65–69 | 3,000,900 | 0.49150 | | 8,708,000 | 0.01379019 | 0.0307630 | 0.0084062 | 0.0531197 |
| 70–74 | 2,771,400 | 0.31775 | | 8,686,000 | 0.02731764 | 0.0307630 | 0.0084062 | 0.0531197 |
| 75–79 | 2,771,400 | 0.17399 | | 7,242,000 | 0.0546802 | 0.0307630 | 0.0084062 | 0.0531197 |
| 80–84 | 2,771,400 | 0.08446 | | 5,328,000 | 0.11855349 | 0.0307630 | 0.0084062 | 0.0531197 |
| 85-89 | 2,771,400 | 0.03041 | | 3,611,000 | 0.24277236 | 0.0307630 | 0.0084062 | 0.0531197 |
| Forgone wages: Point estimates | | | **32,364,103,250** | | | | | |
| Forgone wages: Lower | | | 8,843,712,406 | | | | | |
| Forgone wages: Upper | | | 55,884,388,890 | | | | | |

Note: Functional limitation rate denotes the proportion of the population with certification of levels 2–5 long-term care needs. As the rate in those aged 60–64 was not available, the same proportion as that of the population aged 65–69 was used; As the statistics about detailed wages for those aged 70 or over were not available, we used the same value for older age groups as that of the workers aged 70 or over; Lower and Upper denote lower/upper bounds of 95% confidence interval, estimated in Table 2.

Appendix Table A-9. Per-capita annual costs for medical and long-term care (Unit of currency: JPY)

|  | Certified for support need | | Certified for LTC need | | | | | Total population |
| --- | --- | --- | --- | --- | --- | --- | --- | --- |
| Age | Level 1 | Level 2 | Level 1 | Level 2 | Level 3 | Level 4 | Level 5 |  |
| 65-69 | 297,000 | 437,500 | 1,280,100 | 1,633,100 | 2,550,700 | 3,132,400 | 3,756,600 | 8,708,000 |
|  | (0.4%) | (0.4%) | (0.5%) | (0.5%) | (0.3%) | (0.3%) | (0.3%) |  |
| 70-74 | 286,100 | 413,300 | 1,296,600 | 1,661,000 | 2,575,500 | 3,186,300 | 3,803,800 | 8,686,000 |
|  | (0.9%) | (0.9%) | (1.1%) | (1.0%) | (0.7%) | (0.6%) | (0.5%) |  |
| 75-79 | 269,500 | 392,700 | 1,320,600 | 1,738,900 | 2,694,300 | 3,292,800 | 3,874,100 | 5,689,000 |
|  | (2.3%) | (2.0%) | (2.5%) | (2.0%) | (1.4%) | (1.2%) | (0.9%) |  |
| 80-84 | 269,200 | 387,500 | 1,380,100 | 1,877,600 | 2,867,500 | 3,443,500 | 3,972,400 | 5,328,000 |
|  | (4.8%) | (4.3%) | (5.8%) | (4.3%) | (3.0%) | (2.6%) | (2.0%) |  |
| 85-89 | 290,300 | 408,600 | 1,484,600 | 2,036,800 | 3,018,100 | 3,563,400 | 4,031,000 | 3,611,000 |
|  | (7.1%) | (7.2%) | (10.7%) | (8.5%) | (6.2%) | (5.6%) | (4.0%) |  |
| 90+ | 326,400 | 440,300 | 1,600,700 | 2,159,300 | 3,092,800 | 3,607,300 | 4,025,000 | 2,309,000 |
|  | (5.7%) | (7.4%) | (13.8%) | (13.9%) | (12.6%) | (13.1%) | (8.9%) |  |
| Additional  medical care costs | 102,004 | 204,008 | 306,012 | 385,380 | 447,888 | 485,568 | 610,044 |  |

Note: LTC represents long-term care; Values represent average yearly costs of long-term care per person in each support/care category, with the proportion of those certified among the total population of the same age group in parentheses; Costs include benefits by the insurance, public expenditures, and out-of-pocket payments; Additional medical care costs represent mean differences in age-adjusted healthcare costs between non-certified individuals and those in each support and care category. As medical care costs of people certified for support needs at levels 1 and 2 were not available, these values were imputed by linear interpolation from medical care costs between those without certification and with certification of the level 1 LTC need.

Appendix Table A-10. Additional medical and long-term care costs (Unit of currency: JPY)

|  | Certified for LTC need | | | | |
| --- | --- | --- | --- | --- | --- |
|  | Level 2 | Level 3 | Level 4 | Level 5 | Total |
| 65–69 | 49,070,084,700 | 59,092,192,350 | 66,320,733,750 | 73,331,251,850 | 247,814,262,650 |
|  | (1,129,450) | (2,047,050) | (2,628,750) | (3,252,950) |  |
| 70–74 | 99,213,884,000 | 121,751,424,500 | 135,676,402,400 | 141,372,734,400 | 498,014,445,300 |
|  | (1,162,000) | (2,076,500) | (2,687,300) | (3,304,800) |  |
| 75–79 | 140,430,745,017 | 170,315,198,076 | 187,972,911,094 | 180,570,334,961 | 679,289,189,148 |
|  | (1,243,200) | (2,198,600) | (2,797,100) | (3,378,400) |  |
| 80–84 | 314,212,008,000 | 374,686,704,000 | 407,304,314,400 | 361,367,604,000 | 1,457,570,630,400 |
|  | (1,368,400) | (2,358,300) | (2,934,300) | (3,463,200) |  |
| 85–89 | 456,732,946,350 | 556,740,125,550 | 608,164,058,600 | 500,348,940,875 | 2,121,986,071,375 |
|  | (1,490,925) | (2,472,225) | (3,017,525) | (3,485,125) |  |
| 90 or over | 502,645,163,650 | 730,052,314,500 | 909,028,510,650 | 704,578,508,200 | 2,846,304,497,000 |
|  | (1,567,450) | (2,500,950) | (3,015,450) | (3,433,150) |  |
| Medical care costs | 251,054,409,624 | 243,215,648,685 | 255,513,466,933 | 256,451,906,992 | 1,006,235,432,234 |
|  | (232,374) | (294,882) | (332,562) | (457,038) |  |
| Total additional medical and long-term care costs generated by functional limitations | | |  | **8,857,214,528,107** | |

Note: Additional costs are calculated, based on Appendix Table A-7; Values represent total additional costs in each category, with per-capita additional costs in parentheses.

Appendix Figure A-1. Estimated foregone wages due to functional limitation among people in their 60s


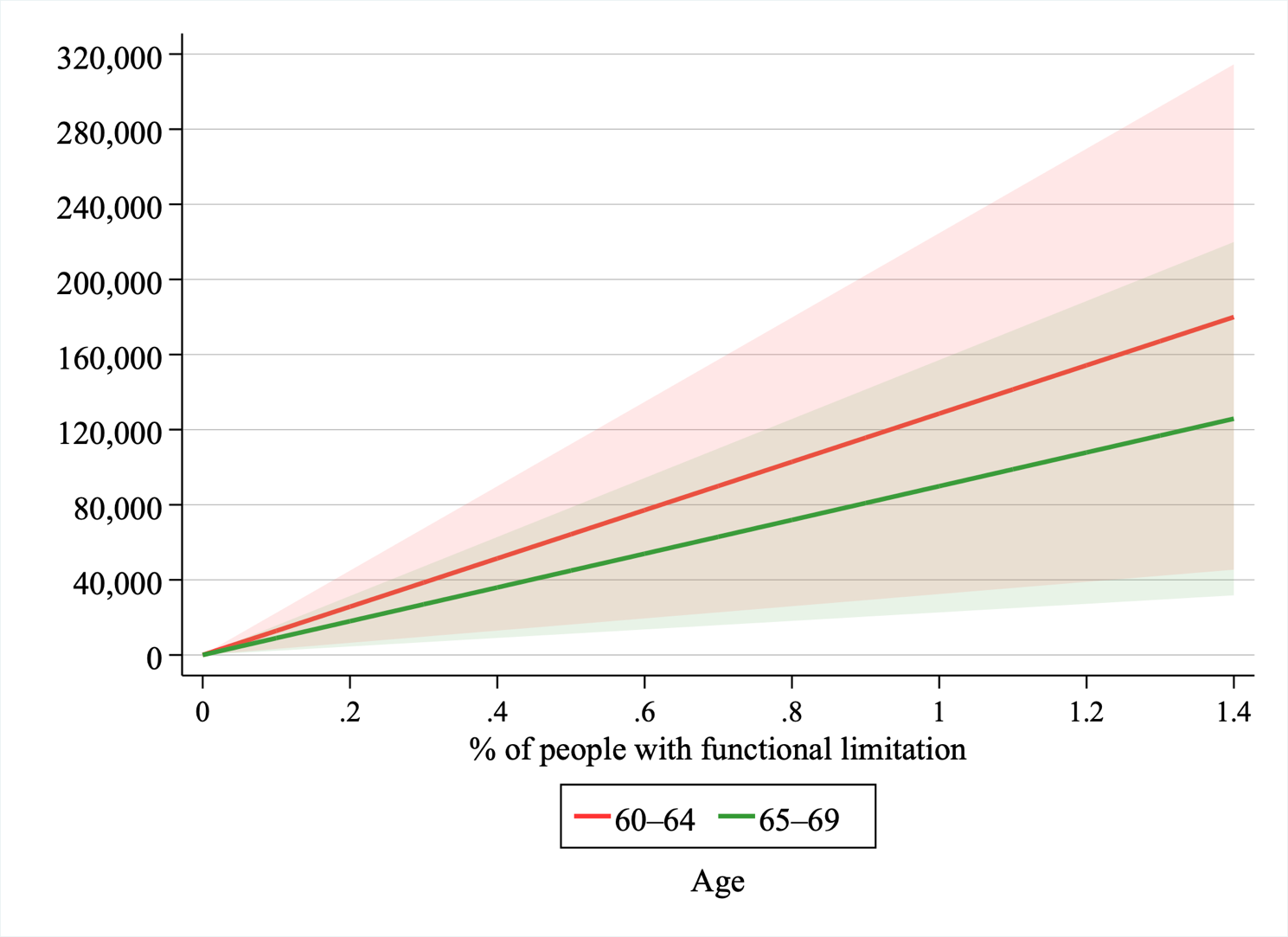


Note: % of people with functional limitation represents the proportion of the population with certification of levels 2–5 long-term care needs. Based on the estimates in Appendix Table A-3, the lines represent foregone wages calculated using the point estimate for the probability of retirement due to functional limitation, with the shaded area representing the 95% confidence interval.
